# Supplementary material for: B4GALT1 as a New Biomarker of Idiopathic Pulmonary Fibrosis
Source: Int J Mol Sci. 2022 Nov 30;23(23):15040. doi: 10.3390/ijms232315040 (PMC9738382; doi:10.3390/ijms232315040)
Supplement: Supplementary file 1 [file ijms-23-15040-s001.zip › Table S1.pdf]

Genes Involved in EMT pathway

ABI3BP	CXCL1	GPX7	MMP14	SERPINH1
ACTA2	CXCL12	GREM1	MMP2	SFRP1
ADAM12	CXCL6	HTRA1	MMP3	SFRP4
ANPEP	CCN1	ID2	MSX1	SGCB
APLP1	DAB2	IGFBP2	MXRA5	SGCD
AREG	DCN	IGFBP3	MYL9	SGCG
BASP1	DKK1	IGFBP4	MYLK	SLC6A8
BDNF	DPYSL3	IL15	NID2	SLIT2
BGN	DST'ECM1	IL32	NNMT	SLIT3
BMP1	ECM2	IL6	NOTCH2	SNAI2
CADM1	EDIL3	CXCL8	NT5E	SNTB1
CALD1	EFEMP2	INHBA	NTM	SPARC
CALU	ELN	ITGA2	OXTR	SPOCK1
CAP2	EMP3	ITGA5	PCOLCE	SPP1
CAPG	ENO2	ITGAV	PCOLCE2	TAGLN
CD44	FAP	ITGB1	PDGFRB	TFPI2
CD59	FAS	ITGB3	PDLIM4	TGFB1
CDH11	FBLN1	ITGB5	PFN2	TGFBI
CDH2	FBLN2	JUN	PLAUR	TGFBR3
CDH6	FBLN5	LAMA1	PLOD1	TGM2
COL11A1	FBN1	LAMA2	PLOD2	THBS1
COL12A1	FBN2	LAMA3	PLOD3	THBS2
COL16A1	FERMT2	LAMC1	PMEPA1	THY1
COL1A1	FGF2	LAMC2	PMP22	TIMP1
COL1A2	FLNA	P3H1	POSTN	TIMP3
COL3A1	FMOD	LGALS1	PPIB	TNC
COL4A1	FN1	LOX	PRRX1	TNFAIP3
COL4A2	FOXC2	LOXL1	PRSS2	TNFRSF11B
COL5A1	FSTL1	LOXL2	PTHLH	TNFRSF12A
COL5A2	FSTL3	LRP1	PTX3	TPM1
COL5A3	FUCA1	LRRC15	PVR	TPM2
COL6A2	FZD8	LUM	QSOX1	TPM4
COL6A3	GADD45A	MAGEE1	RGS4	VCAM1
COL7A1	GADD45B	MATN2	RHOB	VCAN
COL8A2	GAS1	MATN3	SAT1	VEGFA
COMP	GEM	MCM7	SCG2	VEGFC
COPA	GJA1	MEST	SDC1	VIM
CRLF1	GLIPR1	MFAP5	SDC4	WIPF1
CCN2	COLGALT1	MGP	SERPINE1	WNT5A
CTHRC1	GPC1	MMP1	SERPINE2	
