# Supplementary figures and images for: B4GALT1 as a New Biomarker of Idiopathic Pulmonary Fibrosis
Source: Int J Mol Sci. 2022 Nov 30;23(23):15040. doi: 10.3390/ijms232315040 (PMC9738382; doi:10.3390/ijms232315040)

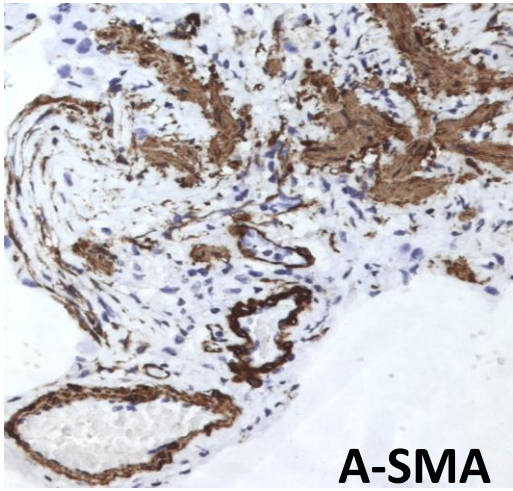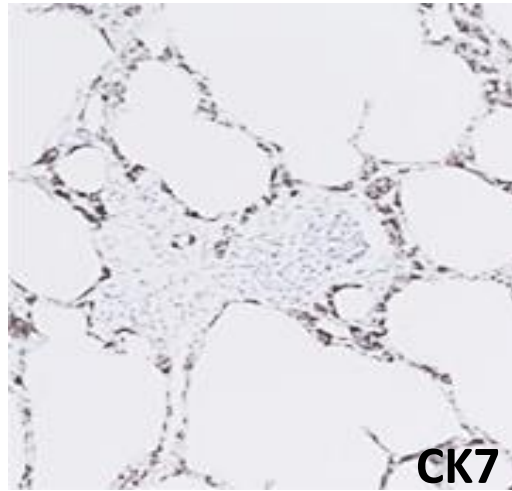

Supplement: Supplementary file 1 [file ijms-23-15040-s001.zip › Figure S1.pdf]

Figure S2

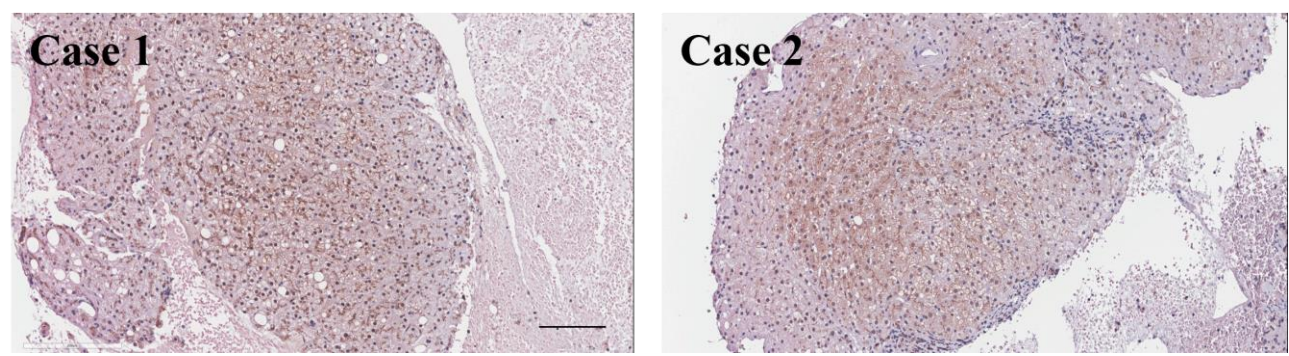

Supplement: Supplementary file 1 [file ijms-23-15040-s001.zip › Figure S2.pdf]
